# Identification of Foot Pathologies Based on Plantar Pressure Asymmetry

**DOI:** 10.3390/s150820392

**Published:** 2015-08-18

**Authors:** Linah Wafai, Aladin Zayegh, John Woulfe, Syed Mahfuzul Aziz, Rezaul Begg

**Affiliations:** 1 College of Engineering and Science, Victoria University, Melbourne VIC-3032, Australia; E-Mails: Linah.Wafai@live.vu.edu.au (L.W.); Aladin.Zayegh@vu.edu.au (A.Z.); 2 Boronia Podiatry, Melbourne VIC-3155, Australia; E-Mail: John.Woulfe@nwhcn.org.au; 3 School of Engineering, University of South Australia, Mawson Lakes SA-5095, Australia; E-Mail: mahfuz.aziz@unisa.edu.au; 4 Gait and Balance Research Group, Institute of Sport, Exercise and Active Living (ISEAL), Victoria University, Melbourne VIC-3032, Australia

**Keywords:** foot pathology, gait symmetry, plantar pressure

## Abstract

Foot pathologies can negatively influence foot function, consequently impairing gait during daily activity, and severely impacting an individual’s quality of life. These pathologies are often painful and correspond with high or abnormal plantar pressure, which can result in asymmetry in the pressure distribution between the two feet. There is currently no general consensus on the presence of asymmetry in able-bodied gait, and plantar pressure analysis during gait is in dire need of a standardized method to quantify asymmetry. This paper investigates the use of plantar pressure asymmetry for pathological gait diagnosis. The results of this study involving plantar pressure analysis in fifty one participants (31 healthy and 20 with foot pathologies) support the presence of plantar pressure asymmetry in normal gait. A higher level of asymmetry was detected at the majority of the regions in the feet of the pathological population, including statistically significant differences in the plantar pressure asymmetry in two regions of the foot, metatarsophalangeal joint 3 (MPJ3) and the lateral heel. Quantification of plantar pressure asymmetry may prove to be useful for the identification and diagnosis of various foot pathologies.

## 1. Introduction

**Figure 1:**
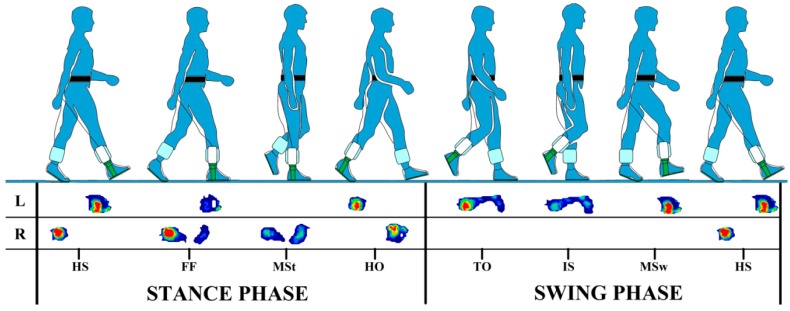
An example of in-shoe plantar pressure measurement during the major phases and events of a full gait cycle (right heel strike to right heel strike). HS = heel strike, FF = foot flat, MSt = midstance, HO = heel off, TO = toe off, IS = initial swing, MSw = midswing.

Gait is one of the most frequently used forms of human movement during daily activity. As an inherently complex task, human gait requires the coordination of both neural and musculoskeletal systems to provide balance and stabilization of the body during movement. Gait is represented by out of phase leg movement, in which each leg successively shifts from one phase of the gait cycle to the next. Analysis of gait parameters plays an important role in the evaluation and characterization of able-bodied and pathological gait. The analysis of foot function in particular is essential, as the feet are the main point of support during gait, and are constantly adapting to various environments and regular exposure to large forces. The gait cycle is fundamentally divided into stance and swing phases. The stance phase accounts for 60% of the total gait cycle, during which the foot is in contact with the ground and bears the full weight of the body. The swing phase comprises the remaining 40% of the gait cycle and begins at the toe off of the foot. During this phase the foot is off the ground and swinging forward to begin the next stance, while the body weight is transferred to the other foot (Figure 1) [1,2].

Changes to normal foot function can result in impaired gait. One of the most common and debilitating clinical conditions that can impact foot function during the gait cycle is the pain pertaining to the pathological foot. This pain is often associated with higher than normal plantar pressure, which can incite various injuries during daily activity. The alteration of normal foot function can instigate a chain of unfavorable outcomes which range from inflicting further high pressure onto a new location of the foot, to placing the individuals at a greater risk of imbalance and injury when walking [3]. These outcomes can also lead to excessive changes in the degree of asymmetry between the plantar pressure of the left and right feet during gait, further increasing the risk of plantar injury. Kinetic measures such as the plantar pressure distribution of the foot can provide valuable information about the nature of an individual’s gait. This can aid in the identification of individuals who may be at risk of developing, or further worsening, a plantar surface injury due to high plantar pressure [4,5].

There have been substantial advancements in plantar pressure measurement over the recent decades. The development of measurement technology has been heavily influenced by an interest in understanding the pressure generated by the feet during human locomotion, both qualitatively and quantitatively [6,7]. Information from these measurements has provided essential support in the assessment of various foot pathologies, including rheumatoid arthritis [8,9], Parkinson’s disease [10,11], and diabetes [12].

Plantar pressure sensing technology has, without doubt, become an indispensable tool for gait analysis in both clinical and research settings. The sensing technology, which is capable of measuring static and dynamic pressure, has played a crucial role in understanding the effects of the pressure on the foot. A variety of pressure sensing systems are currently available in the market, with research continually working towards improving and developing new systems to meet the growing demands of a large user base. There are slight variations in the nature of the pressure sensors used in commercially available systems, and all have advantages and disadvantages in their application. These sensors do, however, share a commonality in that they produce electrical signals that are proportional to the vertical forces acting on the individual sensors when the plantar surface of the foot makes contact with a supporting surface [13,14]. The most common pressure sensors are capacitive, piezoelectric, piezoresistive and resistive sensors. The pressure sensing technology utilizing these sensors can be primarily categorized into platform systems and in-shoe systems [15].

Platform systems are comprised of a large number of sensors arranged in a matrix formation, and generally offer a high spatial resolution for the measurement of plantar pressure [13]. These systems are often embedded into the floor or walkway, and are commonly used in the analysis of barefoot pressure. The use of these platform systems is typically limited to research settings within dedicated laboratory spaces. Obtaining a reproducible walking pattern also necessitates familiarization with the system. As such, there are restrictions on the type of research that can be carried out using platform systems [15,16].

In-shoe pressure measurement systems include sensing elements in the insoles [13,15] and have gained an advantage over platform systems due to their portability. This has facilitated numerous out-of-laboratory research and clinical analysis. One of the greatest advantages of the in-shoe systems is the ability to measure and analyze sequential steps, as the foot typically remains aligned to the same sensors. Reliable pressure measurement can be affected by both the movement of the insoles and the movement of the feet within the shoes. An insole with minuscule spacing between the sensing elements is, however, thought to be more robust to foot movement. The availability of in-shoe systems has not only allowed for the assessment of gait patterns (Figure 1), but has also facilitated evaluation and improvement of footwear and athletic training [17,18]. There has also been a vast interest in applying in-shoe plantar pressure distribution patterns to aid in the production of custom footwear and orthotics for the offloading of high plantar pressure in the foot [4,19].

While measurements of plantar pressure are relatively easy to obtain, quantifying and interpreting the results has proven to be a difficult task, which has consequently limited the analysis and diagnosis of plantar pressure abnormalities [14]. Another challenge in the assessment of plantar pressure is the unreliability of direct feedback from individuals regarding problematic areas of the foot, as these individuals can be either unaware of, or unable to effectively communicate about the problem [20,21,22]. Individuals affected by diabetic neuropathy are of particular concern when it comes to location-specific foot injury, as the plantar surface of their feet often has diminished sensation, or in severe cases, no sensation at all. This means that the onset of foot injury is likely to go undetected, heightening the chances that these individuals will develop a foot ulcer, which in turn increases the likelihood that they will expose the plantar site of injury to further damage due to high pressure during daily activity [21,23,24].

Lower limb symmetry has often been used as a reference to determine able-bodied gait [25]. Various methods have been proposed for the quantification of asymmetry [26,27,28]. The symmetry index (SI) [27] has been used extensively, and is among the most common approaches utilized to analyze gait asymmetry due to its simplicity. However the SI is not without disadvantages, as it requires normalization to a reference value, and can be prone to artificial inflation of calculated asymmetry, especially when the assigned reference value is considerably smaller than the difference between the limbs being examined [29]. In an effort to address these problems, Zifchock *et al.* [28] proposed the symmetry angle (SA) as a more robust method to quantify asymmetry. The SA is an arctan function of the ratio of the left and right limbs, and does not require the need to select a reference value. Although SA values are typically much lower than those of the SI, both the SA and SI values are highly correlated, with the SA having an added benefit of a standard scale (±100%) for the interpretation of the calculated asymmetry [28].

Opinions are divided on what constitutes a diagnosis of pathology due to asymmetrical limb function, and there is no general consensus on the presence, or degree, of lower limb asymmetry in healthy populations [25]. It is essential to understand if asymmetry exists, and the extent to which it exists, in the plantar pressure distribution of the foot during gait. This paper therefore aims to establish a normal range of plantar pressure asymmetry, and to investigate the effect of foot pathologies on deviations from this normal range of asymmetry during gait. As both the SI and SA have been used to quantify the level of asymmetry during gait, this paper will assess both methods and examine the suitability of their application in identifying plantar pressure asymmetry. The paper is structured as follows. Section 2 of the paper details the methodology used for data collection and analysis. The findings of this study are interpreted in Section 3, and discussed in Section 4.

## 2. Experiment and Analysis Methods

### 2.1. Data Collection

Fifty one participants (31 from a control/healthy population and 20 from a pathological population) took part in the data collection. Table 1 shows the participant characteristics, with values presented as mean ± standard deviation. The pathological population were suffering from painful areas on the plantar surface of the foot/feet (with or without accompanying hyperkeratotic lesions) for which the major causative factor is faulty lower limb alignment and foot function. Informed consent was obtained from all participants in accordance with the procedure approved by Victoria University Human Research Ethics Committee. The dynamic plantar pressure distribution data were collected during the participants’ preferred walking speed using the F-scan in-shoe pressure measurement system (Tekscan, MA, USA). The F-scan sensors for each foot provide a resolution of 3.9 sensels per cm^2^ and contain a total of 960 sensing elements, which can be trimmed to fit into the participants’ shoes. Sensors were not used for more than five sessions of data collection. The F-scan software accompanying the pressure measurement system was used to calibrate the sensors according to each participant’s body weight prior to data collection, and record approximately six to seven steps (stances) per foot at 100 Hz for each participant.

**Table 1:** Participant characteristics.

	Control		Pathological	
**Gender**	8 Female	23 Male	14 Female	6 Male
**Age (years)**	34.6 ± 10.2	36.7 ± 9.7	31.4 ± 14.0	41.3 ± 10.7
**Mass (kg)**	63.5 ± 13.2	77.3 ± 13.7	62.9 ± 6.6	82.3 ± 15.2

### 2.2. Data Extraction and Analysis

**Figure 2:**
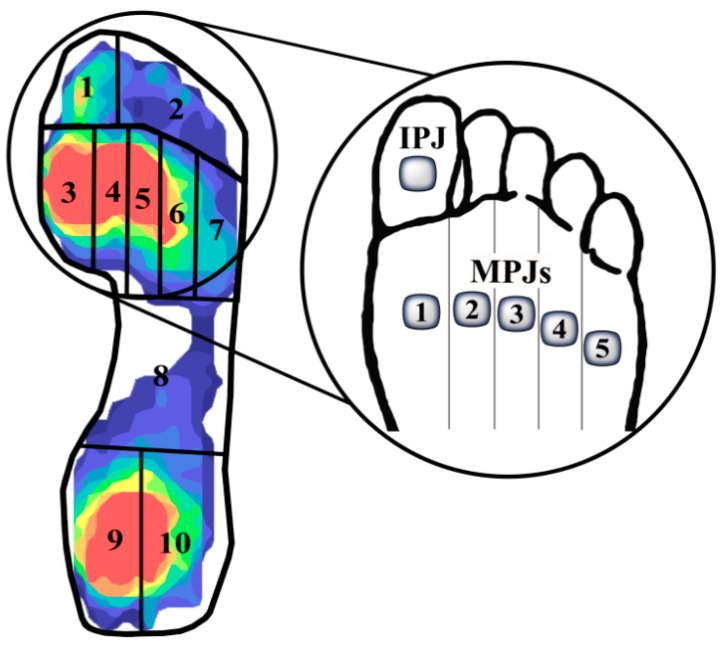
Mask of the 10 anatomical regions of interest in the foot. The magnified area highlights the joints at which pressure values were extracted from regions 1, 3–7.

A customized mask comprised of 10 regions of interest was fitted to each pedobarographic image using the F-scan software. Figure 2 shows an example of the region locations which correspond to (1) interphalangeal joint (IPJ); (2) lesser toes; (3) metatarsophalangeal joint 1 (MPJ1); (4) MPJ2; (5) MPJ3; (6) MPJ4; (7) MPJ5; (8) midfoot; (9) medial heel; and (10) lateral heel. Peak plantar pressure values were extracted from a 2 × 2 analysis box (a 1 cm^2^ area; average of four sensors) within each region during the middle four stances taken by the participants. Peak plantar pressure for the whole foot was also extracted per stance. To accommodate for variations in the shoes among participants, extracted pressure values from each of the 10 regions were normalized to the peak pressure of the whole foot per stance. Normalized data from the four stances were averaged for the left and right feet individually for the 10 regions. These averages were used for the quantification of asymmetry.

### 2.3. Quantification of Asymmetrical Pressure Parameters

The Symmetry Index (SI) [27] and Symmetry Angle (SA) [28] were used to assess and identify the degree of asymmetry between the left and right foot at each of the ten pressure locations (see Figure 2):

Symmetry Index:

(1) $$\text{SI = }\frac{{\text{(X}}_{\text{1}}\;{\text{- X}}_{\text{2}}\text{)}}{\frac{\text{1}}{\text{2}}{\text{(X}}_{\text{1}}{\text{ + X}}_{\text{2}}\text{)}}\text{ × 100\%}$$

Symmetry Angle:

(2) $$\text{SA = }\frac{\left(\text{45° - arctan}\left({\text{X}}_{\text{left}}/{\text{X}}_{\text{right}}\right)\right)}{\text{90}{}^{\circ}}\text{ × 100\%}$$

However, if $\left(\text{45° - arctan}\left({\text{X}}_{\text{left}}/{\text{X}}_{\text{right}}\right)\right)\text{ > 90}{}^{\circ}$, the following equation was used as a substitute:

(3) $$\text{SA = }\frac{\left(\text{45° - arctan}\left({\text{X}}_{\text{left}}/{\text{X}}_{\text{right}}\right)\text{ - 180}{}^{\circ}\right)}{\text{90}{}^{\circ}}\text{ × 100\%}$$

where X_1_ and X_right_ are the pressure parameters for the right foot, and X_2_ and X_left_ are the pressure parameters for the left foot. Perfect symmetry between the feet is signified by a value of 0% for both the SI and SA, while perfect asymmetry is indicated by a value of 100% only for the SA. In this paper, group results have not taken into consideration which foot contains the highest asymmetry, as this result is subjective. The focus has instead been placed on identifying the degree of asymmetry per pressure location, therefore only absolute values will be reported.

### 2.4. Statistical Analysis

All statistical analyses were performed using SPSS Statistics version 20 (SPSS, Inc., Chicago, IL, USA). Prior to analysis, all data were assessed for normality. As data were not normally distributed, the control and pathological groups were assessed using the nonparametric Mann-Whitney *U* test. Data are presented as medians unless otherwise stated. Results from the test were considered to be statistically significant at *p* < 0.05.

As symmetry calculations often use mean values from left and right limbs, there is the possibility that a large amount of intra-limb variability could result in large, but not significant, differences in the mean values, consequently leading to misleadingly large asymmetry values [30]. For a significant measure of asymmetry, it has been proposed that the between-limb difference should be larger than the within-limb difference [30,31]. Therefore, intra-limb variability was assessed for each individual participant, per region of interest, using paired-samples *t*-tests to determine significant differences between the left and right feet. Variables with a *p* < 0.05 were considered to display significant asymmetry, and were reanalyzed using the Mann-Whitney *U* test described above to ascertain whether group asymmetry results were influenced by large individual participant asymmetry values that were not significant [30].

The reliability of the plantar pressure measurement was assessed on five healthy individuals from the control group in two stages: (1) within-session; and (2) between-session. The first stage compared data from two consecutive walking trials from the same session, collected and extracted using the methods described above. The walking trial was repeated one week later for between-session comparison using the same F-scan sensors. The intraclass correlation coefficient (ICC; type 3, k) was calculated for all anatomical regions of interest using measures of absolute agreement. In line with the suggestions of Portney and Watkins [32], ICC values greater than 0.75 indicate good reliability, values between 0.50 and 0.75 show moderate reliability, and values below 0.50 have poor reliability. The relationship between the SI and SA equations was tested using the Spearman’s rank-order correlation.

## 3. Results

### 3.1. Reliability of the Plantar Pressure Measurement

The ICC for within-session walking trials demonstrated excellent reliability, with all regions of interest obtaining ICC values greater than 0.86. Between-session results also showed good reliability, with all regions, apart from the lesser toes and midfoot, achieving ICC values greater than 0.80 (Table 2). These results indicated that the plantar pressure could be measured consistently.

**Table 2:** Within-session and between-session intraclass correlation coefficients (ICCs) for the left and right feet.

Region	Within-Session ICC		Between-Session ICC	
	Left Foot	Right Foot	Left Foot	Right Foot
**IPJ**	0.92	0.96	0.86	0.81
**Lesser toes**	0.92	0.94	0.79	0.78
**MPJ1**	0.91	0.93	0.88	0.83
**MPJ2**	0.96	0.92	0.89	0.92
**MPJ3**	0.92	0.92	0.95	0.95
**MPJ4**	0.88	0.88	0.86	0.87
**MPJ5**	0.88	0.86	0.85	0.84
**Midfoot**	0.90	0.94	0.75	0.76
**Medial heel**	0.94	0.94	0.95	0.96
**Lateral heel**	0.94	0.95	0.94	0.92

### 3.2. Plantar Pressure Asymmetry

**Figure 3:**
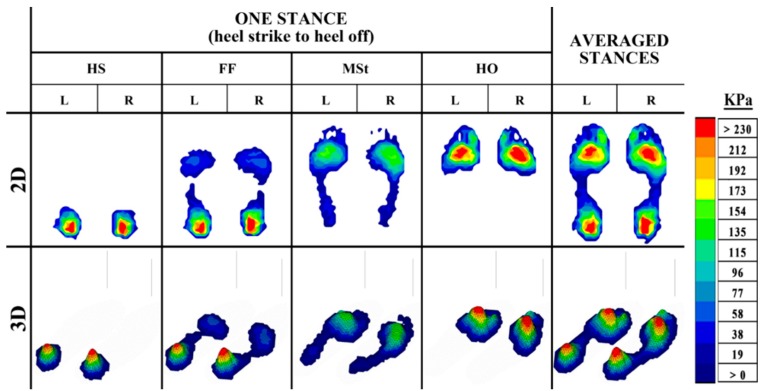
The 2D and 3D representation of in-shoe plantar pressure distribution in a healthy control during the stance phase of the gait cycle, from HS to HO, and the averaged pressure distribution from four stances.

**Figure 4:**
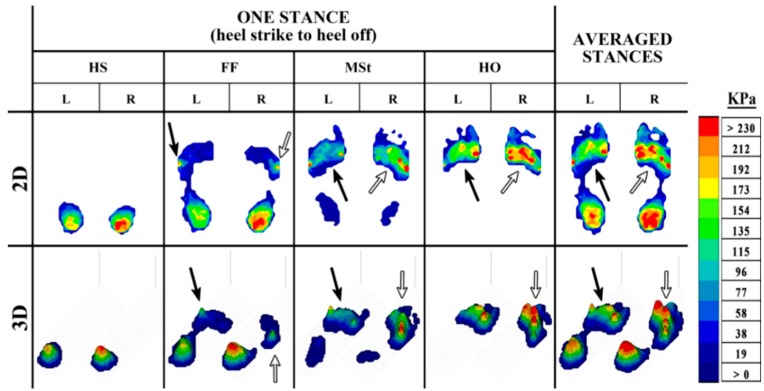
The 2D and 3D representation of an in-shoe plantar pressure distribution resulting from metatarsalgia in the pathological foot during the stance phase of the gait cycle, from HS to HO, and the averaged pressure distribution from four stances.

**Figure 5:**
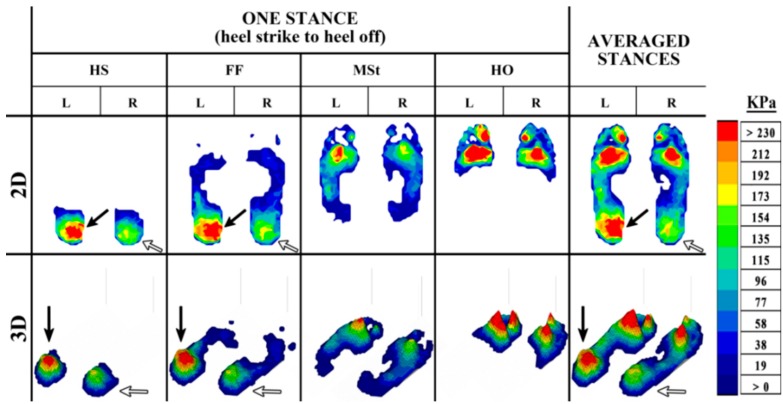
The 2D and 3D representation of an in-shoe plantar pressure distribution resulting from subtalar joint and heel pain in the pathological foot during the stance phase of the gait cycle, from HS to HO, and the averaged pressure distribution from four stances.

The outputs from plantar pressure measurement systems can provide important visual feedback during the diagnostic process to assist in identifying the problem areas of the foot. These outputs, which can be examined in both 2-dimensional (2D) and 3-dimensional (3D) form, give an insight into the progressive changes in pressure during gait. These changes in pressure can be distinguished by a color legend ranging from dark blue (low pressure) to red (high pressure) (Figure 3, Figure 4 and Figure 5).

The pressure changes caused by the rollover of the foot during the stance phase of able-bodied gait (Figure 3) typically follow a similar pattern, with higher pressure in the heel at HS and FF as the body weight is loaded on the heel. As the foot progresses through the stance, the body weight is distributed over the whole foot, allowing for a lower, and more even distribution of pressure at MSt. Following through the final events of a stance, the body weight is transferred to the forefoot during HO, resulting in higher pressure in this region as the MPJs and toes prepare to propel the body forward. Deviations from a normal foot pressure distribution during gait are common in the pathological foot. Asymmetry of the plantar pressure between the feet can be observed, for instance, in cases such as metatarsalgia (Figure 4), and subtalar joint and heel pain (Figure 5).

Metatarsalgia, which is pain at the metatarsals, can cause significant changes in pressure around the affected area. In the example presented in Figure 4, the individual is affected by pain at MPJ4-5 in the left foot. Although asymmetry is apparent during the initial events of the stance, both left and right feet have higher pressure at MPJ5 at FF (black and white arrows). The asymmetry caused by this foot pathology is evident during both MSt and HO of the stance phase, and also when the stances are averaged. During these events, the smooth and even pressure distribution seen in healthy gait (Figure 3) is absent. Instead, the pressure in the left foot remains centered on MPJ5 before shifting to MPJ1, leaving minimal loading of pressure at the MPJs in between (black arrow), as opposed to the right foot, which shows a more even distribution of pressure across the forefoot (white arrow).

In Figure 5, the individual is affected by pain in the left subtalar joint and heel. In this case, the asymmetry caused by the foot pathology is apparent throughout the stance phase, with a much higher level of pressure in the left heel (black arrow) than the right (white arrow). Pressure in the forefoot is also slightly higher in the left foot, although pressure seems to be fairly evenly distributed during the rollover of the foot. These visual observations can be insightful to identify the problem areas, nevertheless a more detailed examination of the results is a necessity in increasing the understanding of the impact of a foot pathology on plantar pressure asymmetry.

Asymmetry levels between left and right feet were determined from the normalized peak plantar pressure parameters at 10 anatomical regions of interest for the control and pathological populations. Asymmetry values from the SI and SA were found to be perfectly correlated based on the Spearman’s rank-order correlation analysis. The degree of plantar pressure asymmetry in the control group showed a median range between ~10%–18% for the SI, and ~3%–6% for the SA across the regions of interest. This was slightly higher for the pathological group, with detected levels of asymmetry ranging between ~14%–22% for the SI and ~4%–7% for the SA (Table 3).

A Mann-Whitney *U* test was run to determine if there were differences in asymmetry levels between the control and pathological groups at each of the anatomical regions of interest. Table 3 summarizes the results. A noticeable difference between the two groups is apparent, with a higher level of asymmetry being detected at the majority of the regions in the feet of the pathological population. However, the levels of asymmetry between the feet were found to be statistically significantly higher for the pathological group in comparison to the control group only at MPJ3 (U = 450, z = 2.70, *p* = 0.01) and at the lateral heel (U = 429, z = 2.30, *p* = 0.02).

**Table 3:** Assessment of median asymmetry percentage in control and pathological groups for Mann-Whitney *U* tests per region of interest. Significant results are indicated with an asterisk (*).

Region	Median Asymmetry (%)				Mann-Whitney *U*	*Z*	*p* Value (Two-Tailed)
	Control		Pathological				
	SI	SA	SI	SA			
**IPJ**	12.57	4.00	17.30	5.49	394	1.62	0.11
**Lesser toes**	14.41	4.58	17.05	5.41	358	0.93	0.35
**MPJ1**	17.86	5.67	19.86	6.30	357	0.91	0.37
**MPJ2**	13.34	4.24	14.57	4.63	315	0.10	0.92
**MPJ3**	10.71	3.41	20.58	6.52	450	2.70	0.01 *
**MPJ4**	16.37	5.20	17.25	5.48	368	1.12	0.26
**MPJ5**	12.36	3.93	14.02	4.45	341	0.60	0.55
**Midfoot**	16.29	5.17	22.17	7.01	391	1.56	0.12
**Medial heel**	9.91	3.15	16.89	5.36	372	1.20	0.23
**Lateral heel**	12.81	4.07	16.56	5.26	429	2.30	0.02 *

To take into account intra-limb variability, asymmetry levels between the control and pathological groups at each of the anatomical regions of interest were reanalyzed using the Mann-Whitney *U* test (Table 4) to only include variables displaying significant asymmetry based on the paired-samples *t*-tests run for each individual participant. The filtering of these variables consequently led to a greatly reduced sample size which, due to greater intra-limb variability, comprised only an approximate 18% of the original participant variables at MPJ5. The lowest amount of intra-limb variability, and therefore the greatest number of participant variables demonstrating significant asymmetry (~37%) was at the medial heel.

**Table 4:** Assessment of median asymmetry percentage in control and pathological groups taking into account intra-limb variability for Mann-Whitney *U* tests per region of interest. Significant results are indicated with an asterisk (*).

Region	Median Asymmetry (%)				*n* (/51.%)	Mann-Whitney *U*	*Z*	*p* Value (Two-Tailed)
	Control		Pathological					
	SI	SA	SI	SA				
**IPJ**	27.10	8.58	57.98	17.96	10 (19.61)	15	1.03	0.38
**Lesser toes**	24.13	7.64	36.76	11.57	13 (25.49)	32	1.76	0.09
**MPJ1**	20.52	6.51	55.96	17.33	11 (21.57)	19	1.43	0.19
**MPJ2**	25.19	7.98	20.68	6.56	17 (33.33)	35	0.00	1.00
**MPJ3**	21.06	6.68	34.62	10.91	13 (25.49)	32	2.16	0.03 *
**MPJ4**	24.82	7.86	43.45	13.62	14 (27.45)	39	1.85	0.07
**MPJ5**	24.75	7.84	61.22	18.90	9 (17.65)	18	2.32	0.02 *
**Midfoot**	24.69	7.82	58.06	17.98	10 (19.61)	21	1.92	0.07
**Medial heel**	23.53	7.45	29.25	9.25	19 (37.25)	64	1.55	0.13
**Lateral heel**	15.11	4.80	30.65	9.68	17 (33.33)	66	2.89	0.002 *

The median asymmetry for both pathological (ranging between ~21%–61% for the SI, and ~7%–19% for the SA) and control (ranging between ~15%–27% for the SI, and ~4%–9% for the SA) groups is higher when accounting for intra-limb variability, as the variables identified as significant by the individual paired-samples *t*-tests typically also had a larger calculated asymmetry percentage. As with the group results, the levels of asymmetry between the feet were also found to be statistically significantly higher for the pathological group in comparison to the control group at MPJ3 (U = 32, z = 2.16, *p* = 0.03) and at the lateral heel (U = 66, z = 2.89, *p* = 0.002). Additionally, MPJ5 also showed a statistically significant increase in the asymmetry level for the pathological group *versus* the control group (U = 18, z = 2.32, *p* = 0.02).

## 4. Discussion

This study aimed to determine a normal range of plantar pressure asymmetry, and to investigate the effect of foot pathologies on deviations from this normal asymmetry range during gait. The results of this study support the notion of asymmetrical plantar pressure during able-bodied gait. This is consistent with previously published research. The findings of this study also showed significant increases in the level of plantar pressure asymmetry at the third MPJ and the lateral heel in a pathological population.

Plantar pressure analysis predominately relies on the detected level of pressure to make a judgment in regards to the presence of a foot pathology. This has eased the diagnostic process involved during foot assessment, and significantly improved the treatment capabilities for the pathological foot, particularly in cases of pressure redistribution to ease foot pain and reduce the risk of plantar injury [4,17,18,33,34,35]. It is also a well-established finding that high plantar pressure is a contributing factor to plantar injury, particularly in individuals with diabetes [5,36,37]. While pressure data is very useful for the diagnosis of foot pathology, generalized assumptions or judgments based solely on the level of measured plantar pressure cannot be made. Measured levels of plantar pressure vary widely between individuals, and often there is no single pressure threshold that is an indicator for the onset of plantar injury. This has been a particularly problematic situation in diabetic foot research; in which there has been an ongoing struggle to clearly identify a pressure threshold that would indicate that an individual is at risk of developing a plantar ulcer [38,39,40,41].

Foot function can be severely affected by injuries to the forefoot, particularly at the metatarsal region. The site of injury is often reflected not only in the plantar pressure distribution, but also in the measures of asymmetry between the feet [42]. The presence of large asymmetry between the feet can be an indication that there has been a notable negative impact to normal foot function and unequal loading of the two feet, as limb function during normal gait is frequently assumed to be symmetrical. This perception is commonly transferred into clinical settings, where the identification of asymmetry can play an important role in the diagnosis of a pathology. Methods to quantify asymmetry during gait, such as the SI, are simple to use and to understand for both clinicians and patients. Their application in clinical settings can provide a quick and easy measure of improvement or decline in affected areas. The restoration of symmetry during gait is therefore often a primary target set for patient rehabilitation [43,44]. The analysis of gait symmetry during the rehabilitation process can aid in monitoring and assessing the effects of treatments or interventions, with improvements in the measured symmetry being used as indications of clinically effective treatment [45].

However, asymmetry in the lower limb is not only associated with the manifestation of a pathology, but is also found to be present in able-bodied gait in this study, with a normal range of asymmetry determined to be between approximately 10%–18% based on the SI, and 3%–6% based on the SA across the regions of interest examined on the plantar surface of the foot. The assumption of symmetry in able-bodied gait can be problematic in gait analysis, especially when lower limb asymmetry is considered to be related to gait pathology [25]. This has long been a point of contention in gait analysis, and the definition of symmetry itself has varied in earlier years from a perfect agreement between the actions of the lower limbs [29,46], to the absence of statistical bilateral limb differences [47]. Currently, a general consensus regarding the presence of lower limb asymmetry in able-bodied gait is still not accepted; however, research in support of asymmetrical limb function has been increasing. Rather than attributing the differences in lower limb function to the ramifications of limb abnormality, asymmetry in able-bodied gait is believed to be a product of the natural functional differences between the roles of the lower limbs. The concept of functional asymmetry relies on the definitions of limb dominance to differentiate between the roles of the lower limbs. The dominant (preferred) limb makes larger contributions towards forward propulsion, while the non-dominant (non-preferred) limb contributes towards support [25,48]. Reasons as to why functional asymmetry exists are still unclear [25,49]. Limb dominance is believed to be task dependent, indicating that the difficulty of the task performed can impact the roles the limbs play in support and propulsion. It has been suggested that the roles of the lower limbs contribute towards a local asymmetry (such as those found in muscle activity and joint kinetics) [50]. Consecutive steps taken during gait are not perfectly repetitive due to the continuous adjustments made by neuro-musculoskeletal systems in an effort to stabilize the body during gait. These minor variations are present between steps regardless of the type of surface used during locomotion [31,51]. However, the behavior of the lower limbs, when considered as a whole, is symmetrical (global symmetry) [48,50]. As such, the existence of local asymmetries may be due to a compensatory mechanism to allow for a global symmetry during gait [50].

This study quantifies measures of asymmetry based on the SI proposed by Robinson *et al.* [27] and the SA proposed by Zifchock *et al.* [28]. As previously noted, SA values are much lower than the values calculated using the SI. Both methods were found to be perfectly correlated in this study, in line with findings from previously published research showing high correlations between the two approaches [28,51,52]. Moreover, both equations seem to provide a good representation of the level of plantar pressure asymmetry between the feet for both able-bodied and pathological gait, although use of the SI may be preferred in clinical applications due to its simplicity. Among other symmetry equations that have been proposed, the outcomes of these symmetry calculations appear to be similar and highly correlated, with no single equation holding an advantage over another [52]. Concerns have been raised about the potential for over- or under-estimation of asymmetry when using a symmetry calculation, as the levels of asymmetry have no statistical basis and are often not comparable across different studies [26]. The potential influence of large intra-limb variability on calculated levels of asymmetry may also be problematic [30]. It has been suggested that inter-limb variability should be larger than intra-limb variability for a variable to be considered statistically significant [30,31]. Intra-limb variability was assessed for individual participants in this study to determine if any large, non-significant asymmetry values were influencing group comparisons. Varying levels of intra-limb variability were present across the regions of interest, with significant asymmetry identified in participant variables that also had a larger calculated asymmetry. This confirmed that individual large, non-significant asymmetry was not affecting group results. The results from the intra-limb variability assessment appear to be in line with the findings that include all participant variables in the group asymmetry comparison, albeit with higher median asymmetry percentages, which may stem from the small sample size for all regions of interest examined. The lack of a standard method for the quantification of asymmetry is an important issue that requires crucial attention. The adoption of a uniform method of calculation of plantar pressure asymmetry has the potential for it to be used across the output of all types of plantar pressure measurement systems, allowing for a greater possibility of study comparison.

Although the findings show significant regional plantar pressure asymmetry, several limitations are present in this study. The pathological population comprised of individuals with varying foot problems, which include hallux valgus, metatarsalgia, and subtalar joint and heel pain. Different patterns of asymmetry are likely to be observed in different foot pathologies during gait. While these findings are not conclusive for all pathologies, there does appear to be a shared commonality in the changes to plantar pressure asymmetry at the third MPJ and also at the lateral heel. Although it should be noted that plantar pressure measurements could potentially be influenced by body mass index (BMI) and also the nature of the shoes worn during gait, both of which were not assessed in this study.

The difference in sample size between the control and pathological population in this study is potentially another limiting factor. The small number of participants in the pathological group did not allow for a more in-depth analysis into the effects of particular pathologies on plantar pressure asymmetry. Further research needs to be performed with a larger sample size to allow for comparisons among subjects with specific types of foot pathologies. Ideally, a large-scale analysis of plantar pressure symmetry should also be carried out in a healthy control population. This is particularly important in the development of a standardized definition of symmetry in the feet, which would provide researchers and clinicians with a predetermined normal range of symmetry, outside of which would indicate abnormality in the plantar pressure distribution.

## 5. Conclusions

This study supports the presence of plantar pressure asymmetry in able-bodied gait. Significant increases in the plantar pressure asymmetry of the pathological population have also been demonstrated. Assessments of plantar pressure asymmetry could potentially assist and improve the diagnostic process to determine the presence of a foot pathology. Furthermore, these assessments may serve to be a useful tool in early detection of plantar injury, and can also aid in the identification of effective treatment options, allowing for more informed decisions to take place in regards to appropriate methods of treatment. Future research would benefit from investigating pressure asymmetries in specific pathologies, including the impact of limb dominance on plantar pressure, and the long-term changes that take place in the presence of a foot pathology during gait.
